# Complications of surgery for gastro-entero-pancreatic neuroendocrine neoplasias

**DOI:** 10.1007/s00423-020-01869-0

**Published:** 2020-04-15

**Authors:** Max B. Albers, Martin Almquist, Anders Bergenfelz, Erik Nordenström

**Affiliations:** 1 Department of Surgery, and Department of Clinical Sciences, Skåne University Hospital, Lund University, Lund, Sweden; 2 Department of Visceral, Thoracic, and Vascular Surgery, Philipps University Marburg, Marburg, Germany

**Keywords:** Neuroendocrine neoplasia, Perioperative complications, Surgery, Gastro-entero-pancreatic system, Small intestine, Pancreas

## Abstract

**Purpose:**

Surgery is recommended for most patients with gastro-entero-pancreatic neuroendocrine neoplasias (GEP-NENs). Rates of complications and perioperative mortality have been reported in few mostly retrospective single-center series, but there has been no detailed analysis on risk factors for perioperative complications and mortality to date.

**Methods:**

Data of patients with GEP-NENs operated between January 2015 and September 2018 were retrieved from EUROCRINE©, a European online endocrine surgical quality registry, and analyzed regarding rate and risk factors of surgical complications. Risk factors were assessed by logistic regression.

**Results:**

Some 376 patients (211 female, 167 male; age median 63, range 15–89 years) were included. Most NENs were located in the small intestine (SI) (*n* = 132) or pancreas (*n* = 111), the rest in the stomach (*n* = 34), duodenum (*n* = 30), appendix (*n* = 30), colon, and rectum (*n* = 22), or with unknown primary (*n* = 15). Of the tumors, 320 (85.1%) were well or moderately differentiated, and 147 (39.1%) of the patients had distant metastases at the time of operation. Severe complications (Dindo-Clavien ≥ 3) occurred in 56 (14.9%) patients, and 4 (1.1%) patients died perioperatively. Severe complications were more frequent in surgery for duodenopancreatic NENs (*n* = 31; 22.0%) compared with SI-NENs (*n* = 15; 11.4%) (*p* = 0.014), in patients with lymph node metastases operated with curative aim of surgery (*n* = 24; 21.4%) versus non-metastasized tumors or palliative surgery (*n* = 32; 12.1%) (*p* = 0.020), and in functioning tumors (*n* = 20; 23.0%) versus non-functioning tumors (*n* = 30; 13.5%) (*p* = 0.042). Complication rates were not significantly associated with tumor stage or grade.

**Conclusions:**

Severe complications are frequent in GEP-NEN surgery. Besides duodenopancreatic tumor location, curative resection of nodal metastases and functioning tumors are risk factors for complications.

## Introduction

Gastro-entero-pancreatic neuroendocrine neoplasias (GEP-NENs) constitute a heterogeneous group of tumors first described in 1907 [1]. Epidemiological data on GEP-NEN has improved understanding of the tumors’ biology, tumor classification, and has facilitated choice of therapy, and treatment strategies during the past years. With a clearer understanding of the tumors and a precise histologic definition [2], the reported crude incidence has risen worldwide and is now approximately 3.5/100.000 [3]. While small intestinal (SI) NEN used to be detected much more frequently than pancreatic (p) NEN, more recently, the diagnosis of pNEN has been almost as frequent as SI-NEN [3, 4]. National and international consensus guidelines for the management of GEP-NEN recommend surgery for most patients [5–12]. Besides surgical resection, peptide receptor radionuclide therapy, hormonal therapy, and cytotoxic chemotherapy play an important role in current treatment algorithms. Since many patients, in particular with G1 tumors, will live for a long time with disease, even without treatment, it is important to balance side effects of treatment versus the benefit of therapy, especially surgery.

Very little data have been published on complications after the surgical resection of GEP-NEN. In the past, mostly single-center and retrospective series reported complication rates of 5–35% depending on the tumor localization and type of operation [13–18]. The most common complication following surgery for pNEN is postoperative pancreatic fistula, delayed gastric emptying, and hemorrhage. Pancreatic fistula is more frequent after enucleations than pancreatic resections and accounts for more than one half of the complications after duodenopancreatic surgery for pNEN [14, 19]. Common complications after resection of SI-NEN comprise hemorrhage, small bowel obstruction, and local infections [16].

The aim of the present study was to determine the frequency and risk factors of severe complications in GEP-NEN surgery.

## Patients and methods

### EUROCRINE database

Data of all patients who underwent surgery for GEP-NEN between January 2015 and September 2018 were retrieved from the prospectively maintained EUROCRINE©-database (http://www.EUROCRINE.com) and retrospectively analyzed. (Ethical approval was given by the Regional Ethical Review Board of Lund University (2018/488). EUROCRINE is a web-based online endocrine surgical quality registry supported by European national endocrine surgical societies and the European Society of Endocrine Surgery (ESES). EUROCRINE aims to decrease morbidity and mortality in endocrine tumors, with a special focus on rare tumors. Some 92 units in Europe are at the moment connected to the registry. In the EUROCRINE database variables reflecting diagnostic processes, indications for surgical treatment, type of surgical procedures, use of resources, tumors’ details, additional therapies, and outcomes are collected.

Neuroendocrine tumors were diagnosed by microscopy and immunohistochemical staining according to the defined criteria [20]. Specific variables for complications were registered, including free text, and graded according to the Dindo-Clavien classification [21]. In case of multiple complications, the grade of the most severe event was given. Data were extracted anonymized. Data from this patient cohort have not been published previously. Patients were excluded from the analysis, if information on tumor localization, surgical procedure, or complications was missing or not conclusive.

All patients received at least minimal preoperative diagnostic examinations as described by ENETS guidelines [7, 8, 11, 22, 23]; additional examinations of the NEN disease and for perioperative risk stratification were performed at the discretion of the treating centers.

Surgical procedures were indicated and performed according to local standards.

### Grading and staging of tumors

During the period of data collection, the WHO grading system for GEP-NEN changed. All information given in the present manuscript are according to the current 2017 WHO definition criteria [2].

Tumors were graded as NET G1, NET G2, NET G3, or NEC G3 by the given mitotic and Ki67-indices as well as cell differentiation, whenever sufficient data was available. In tumors with mitotic index or Ki67 index > 20% and missing or non-conclusive data on tumor differentiation, the tumor grade was referred to as NET/NEC G3.

TNM 8 staging could not be adapted by the given information; therefore, stage was reported by criteria from the time of examination for the analysis within this report.

### Statistics

The retrieved data were transferred to Stata/IC 14.2 for Mac (Stata Corp.; TX, USA) for statistical analysis. Parametric data are presented as mean and standard deviation. Nonparametric data are presented as median and range. *p* values < 0.050 were considered statistically significant. Fisher’s exact test or chi^2^ test was used for crude analysis of risk factors for perioperative complications. Adjusted effects of risk factors on surgical complications were calculated by multivariate logistic regression models and presented as odds ratios (OR) and 95% confidence interval (CI).

## Results

### Patient demographics and tumor characteristics

Some 376 patients underwent surgery for GEP-NEN and were registered in the EUROCRINE© database by 23 centers from 9 different countries. Of these patients, 211 (55.8%) were female and 167 (44.2%) male. The median age at the time of surgery was 63 (range 15–89) years.

**Fig. 1:**
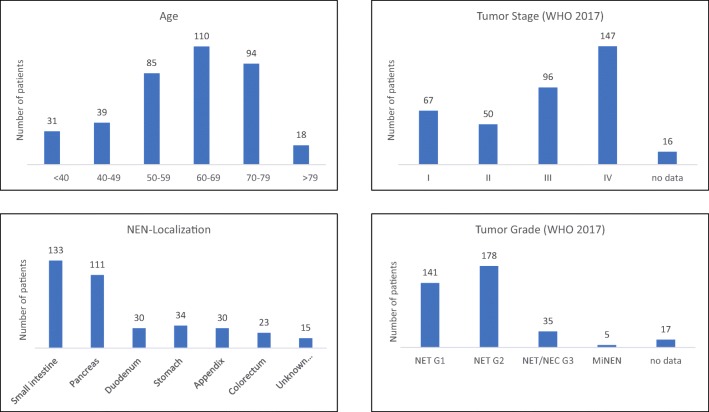
Patient characteristics and tumor details. NEN = neuroendocrine neoplasm

The localization of the NEN was most commonly the small intestine (SI-NEN, *n* = 132, 34.9%), followed by the pancreas (pNEN, *n* = 111, 29.4%), stomach (*n* = 34, 9.0%), duodenum (dNEN, *n* = 30, 7.9%), appendix (n = 30, 7.9%), colon and rectum (*n* = 22, 5.8%), or a distant metastasis with unknown primary (CUP, *n* = 15, 4.0%). Information on tumor grading was available in 361 patients. According to the WHO 2017 classification [2], 141 (39.1%) patients had a NET G1, 180 (49.9%) NET G2, 6 (1.7%) NET G3, 13 (3.6%) NEC G3, and 5 (1.4%) mixed neuroendocrine-non-neuroendocrine neoplasias (MiNEN). In 16 (4.4%) patients, NET G3 and NEC G3 could not be differentiated from the given information. The tumor stage according to the UICC definition that was valid at the time of diagnosis was stage I in 65 (17.2%) patients, stage II in 51 (13.5%) patients, stage III in 96 (25.4%) patients, and stage IV in 147 (39.9%) patients. No data on the tumor stage was available in 21 (5.6%) patients (Fig. 1).

The tumors were functioning in 87 (23.1%) patients, and non-functioning but with reactivity in immunohistochemistry in 95 (25.2%) patients. The primary tumor of hormonally functioning disease was most commonly located in the small intestine (*n* = 42, 37%) or the pancreas (*n* = 34, 36%). Ten patients had multiple endocrine neoplasia type 1, and three patients had other hereditary disease.

### Performed surgery

The aim of the performed operation was curative in 268 (71.3%) patients, palliative in 82 (21.8%) patients, and explorative in 26 (6.9%) patients. The procedure included resection of the primary tumor in 351 (93.4%) patients, selective lymphadenectomy in 77 (21.8%) patients, and systematic lymphadenectomy in 182 (51.6%) patients. Liver metastases were resected in 38 (10.1%) patients.

### Complications

The number of patients with severe complications (Dindo-Clavien ≥III) was 56 (14.9%). According to Dindo-Clavien classification, 43 were grade III, nine were grade IV, and four patients died of complications (grade V). Most patients with severe complications had the primary tumor in the duodenum or pancreas (*n* = 31), followed by the small intestine (*n* = 15), stomach (*n* = 4), colon and rectum (*n* = 3), appendix (*n* = 2), or had no known primary tumor (*n* = 1) (Table 1).

**Table 1:** Postoperative complications by localization of the primary tumor. In patients with multiple complications, only the most severe complication is listed

Primary tumor location	Complications by Dindo-Clavien Classification; *n* (%)			
	0 – II	III	IV	V
Small intestine (*n* = 133)	118 (88.7)	13 (9.8)	1 (0.8)	1 (0.8)
Pancreas (*n* = 111)	88 (79.2)	17 (15.3)	5 (4.5)	1 (0.9)
Duodenum (*n* = 30)	22 (73.3)	5 (16.7)	1 (3.3)	2 (6.7)
Stomach (*n* = 34)	30 (88.2)	3 (8.8)	1 (2.9)	0
Appendix (*n* = 30)	28 (9.3)	2 (6.7)	0	0
Colorectum (*n* = 23)	20 (87.0)	2 (8.7)	1 (4.3)	0
Unknown primary (*n* = 15)	14 (93.3)	1 (6.7)	0	0

Severe complications comprised postoperative hemorrhage (*n* = 18, 4.8%), local infections or abscesses (*n* = 15, 4.0%), pancreatic fistula (*n* = 12, 3.2%; 9.2% of duodenal and pNEN), anastomotic leakage (*n* = 6, 1.6%), deep vein thrombosis (*n* = 5, 1.3%), pulmonary embolism (*n* = 3, 0.8%), and myocardial infarction (*n* = 3, 0.8%). Other severe complications occurred in combination with one of the above (*n* = 16, 4.3%), or alone (*n* = 7, 1.9%). Pancreatic fistula occurred only after resection of duodenal or pNENs; none of the other complications was associated with the localization of the primary tumor.

Using univariable logistic regression (Table 2), the complication rate was significantly higher in surgery for duodenal or pNEN (*n* = 31, 22.0%) compared with small intestinal NEN (*n* = 15, 11.3%) (*p* = 0.014), OR 2.20 (1.13–4.29), or compared with all other primary tumors (*n* = 25, 10.3%) (*p* = 0.003), OR 2.38 (1.34–4.23). In functioning tumors, the complication rate was 23.0% (*n* = 20) and significantly higher than in non-functioning tumors (*n* = 30, 13.5%) (*p* = 0.044), OR 1.91 (1.02–3.59).

**Table 2:** Regression analysis of potential risk factors for severe postoperative complications (Dindo-Clavien ≥ 3)

Risk factor	*n*	Complication rate	versus	*n*	Complication rate	*p* value	OR (95% CI)
Duodenopancreatic primary tumor	131	22%	Small intestinal primary tumor	133	11%	0.014	2.20 (1.13–4.29)
Duodenopancreatic primary tumor	131	22%	All non-duodenopancreatic tumors	245	10%	0.003	2.38 (1.34–4.23)
Curative resection of lymph node metastases	112	21%	No Lymph node metastases or palliative aim of resection	264	12%	0.020	1.99 (1.11–3.56)
Functioning tumor	87	23%	Non-functioning tumors	222	13%	0.042	1.91 (1.02–3.59)
NET/NEC G3	35	11%	NET G1, NET G2	321	16%	0.505	0.69 (0.17–2.09)
Age ≥ 60 years	221	14%	Age < 60 years	155	16%	0.573	0.85 (0.46–1.57)
Cardiovascular disease	112	12%	No cardiovascular disease	264	16%	0.249	0.68 (0.32–1.36)
BMI ≥ 30 kg/m2	59	24%	BMI < 30 kg/m2	193	15%	0.120	1.76 (0.79–3.78)
Men	166	17%	Women	210	13%	0.330	0.75 (0.41–1.39)

*OR* odds ratio; *CI* confidence interval; *NET* neuroendocrine tumor; *NEC* neuroendocrine carcinoma; *BMI* body mass index

Resection of lymph node metastases with curative treatment intention was associated with an increased risk for complications. In these 112 patients, the complication rate was 21.4% (*n* = 24) and significantly higher than in 264 patients without lymph node metastases or palliative treatment intention (*n* = 32, 12.1%) (*p* = 0.021), OR 1.99 (1.11–3.56). Complication rates of 96 patients with tumor stage III (*n* = 21, 21.9%) were higher than of 280 patients with stages I, II, and IV (*n* = 35, 12.5%), OR 1.97 (1.08–3.58).

No association with severe perioperative complications was found for NET/NEC G3 tumors versus NET G1 and G2, age ≥ 60 years versus age < 60 years, cardiovascular disease versus no cardiovascular disease, BMI ≥ 30 versus BMI < 30, or men versus women.

In a multivariable logistic regression model with the dependent variable severe complication (Dindo-Clavien type III or higher) and the independent variables duodenopancreatic primary tumor location, curative resection of lymph node metastases, functioning tumor, independent association was confirmed for duodenopancreatic primary tumors OR 2.40 (1.33–4.70) and for curative resection of lymph node metastases, OR 2.5 (1.33–4.83). In this model, functioning tumors were not independently associated with severe complications, OR 1.86 (CI 0.97–3.56).

## Discussion

In this retrospective study of 376 patients from the prospectively maintained EUROCRINE® database who underwent surgery for GEP-NEN, severe complications (Dindo-Clavien ≥ 3) were observed in 15% of patients. Four patients died perioperatively. The most common complications were bleeding (4.8%), followed by surgical site infections (4.0%) and pancreatic fistula (3.2%). Duodenopancreatic location of the primary tumor, resection of lymph node metastases, and surgery for functioning tumors were associated with a higher rate of complications.

This study focused on the frequency and risk factors of complications of surgical treatment of GEP-NEN, giving an overview on perioperative complications in a large contemporary European multicenter cohort. To provide a comprehensive analysis, primary tumors from the whole gastro-entero-pancreatic system were incorporated and analyzed separately. The studied cohort was well in line with contemporary epidemiological data regarding distribution of primary tumor locations, as well as patients’ characteristics [3].

Comparison to earlier reported findings is hampered by heterogeneous definitions and severity of complications. Most previous studies reported complication rates of surgery for duodenal and pNEN and only one study included loco-regional resection of SI-NEN [17]. Reported rates of severe complications of locoregional resective surgery for SI-NEN were 7.8% [17], and for duodenal and pNEN 17–25% [24–26]. Recently, similar severe complication rates of laparoscopic and robotic distal pancreatic resections of 17–37% have been reported [27, 28]. In 2000, Soreide et al. reported perioperative morbidity in 11% and mortality in 2.6% of 154 patients undergoing surgery for gastrointestinal NEN [29]. Resection of liver metastases or cytoreductive surgery caused complications in 19–44% of patients [15, 30]. In-hospital mortality for pancreatic and gastrointestinal NEN resection was reported to be 3–6% and 0.5–2.6%, respectively [17, 18, 29]. No data on complications of surgery for colorectal NEN could be found in the literature; for colorectal surgery for any indication, rates of severe complications (Dindo-Clavien ≥ 3) up to more than 20% have been reported [31–33].

Male gender, obesity, and comorbidities, among others, have been identified as risk factors for complications in abdominal general surgery and colorectal surgery [33]. However, the unique clinical presentation of NEN with often slow progress, bulky lymph node disease, fibrosis of the mesentery, and hormonal secretion, suggests that a separate evaluation of risk factors for surgical complications in these patients is necessary. However, data on risk factors for adverse events in GEP-NEN surgery are scarce. Jilesen et al. [25] analyzed complications after locoregional resection of pNEN and found a higher risk for tumors of the pancreatic head and for patients with BMI ≥ 25 kg/m^2^. This finding could not be confirmed in the current investigation.

Endocrine function of NEN has been shown to correlate with shorter survival [17], but its effect on postoperative complications has not been studied previously. In the current study, functioning tumors were associated with more complications in the univariable analysis, but not in the adjusted analysis. It remains uncertain, whether hormonally functioning tumors are an independent risk factor per se, or might also be associated with advanced disease and the location of functioning tumors, which was hardly the appendix or stomach, but most commonly the small intestine and the pancreas.

Systematic lymph node dissection is considered technically demanding, especially in pancreatic surgery and in resection of SI-NEN, as the latter often cause bulky mesenterial tumor load and mesenterial fibrosis [7]. The findings of the current study showed that curative surgical treatment in patients with lymph node metastases is independently of the primary tumor site associated with a higher frequency of severe postoperative complications, most commonly bleeding, compared with procedures without curative resection of lymph node metastasis.

Center influence on complications was not possible to assess given the large number of centers and the small number of patients from most centers. Generally, GEP-NEN surgery should be performed at centers with a high level of experience in abdominal endocrine surgery [34].

With the increase of nonsurgical options for the treatment of GEP-NEN, the findings of this study might be taken into consideration in patients with an unclear indication for surgery, especially in palliative settings.

In summary, severe complications are frequent in surgery for GEP-NEN. Tumor location, curative resection of nodal metastases, and functioning tumors are risk factors for complications.
